# Economic benefit evaluation of water resources allocation in transboundary basins based on particle swarm optimization algorithm and cooperative game model—A case study of Lancang-Mekong River Basin

**DOI:** 10.1371/journal.pone.0265350

**Published:** 2022-07-19

**Authors:** Fei Bi, Haiwei Zhou, Min Zhu, Weiwei Wang

**Affiliations:** 1 School of Business, Hohai University, Nanjing, China; 2 International River Research Centre, Hohai University, Nanjing, China; 3 School of Economics & Management, The Open University of China, Beijing, China; Torrens University Australia, AUSTRALIA

## Abstract

The present work aims to find the optimal solution of Nash Equilibrium (NE) in the traditional Game Theory (GT) applied to water resources allocation. Innovatively, this paper introduces Particle Swarm Optimization (PSO) into GT to propose a cooperative game model to solve the NE problem. Firstly, the basic theory of the PSO algorithm and cooperative game model is described. Secondly, the PSO-based cooperative game model is explained. Finally, the PSO-based cooperative game model is compared with the Genetic Algorithm (GA) to test the performance. Besides taking the countries in Lancang Mekong River Basin as the research object, this paper discusses each country’s water consumption and economic benefits under different cooperation patterns. Then, a series of improvement measures and suggestions are put forward accordingly. The results show that the average server occupancy time of the PSO-based cooperative game model is 78.46% lower than that of GA, and the average waiting time is 79.24% lower than that of the GA. Thus, the model reported here has higher computational efficiency and excellent performance than the GA and is more suitable for the current study. In addition, the multi-country cooperation mode can obtain more economic benefits than the independent water resource development mode. This model can quickly find the optimal combination of 16 cooperation modes and has guiding significance for maximizing the benefits of cross-border water Resource Utilization. This research can provide necessary technical support to solve the possible contradictions and conflicts between cross-border river basin countries and build harmonious international relations.

## Introduction

Water is the cradle of living creatures and a vital economic and strategic resource for society [1]. International rivers (cross-border rivers) have caused controversy among the countries and regions they flow through due to the Resource Utilization (RU) issue of water, especially in low flow years [2]. In particular, the Lancang Mekong River, which originates in China and flows through Myanmar, Laos, Thailand, Cambodia, and Vietnam, has caused territorial and resource disputes in the past few decades [3]. The drastic seasonal changes in precipitation and the El Niño phenomenon have led to frequent conflicts over water resources in the Lancang-Mekong River Basin, affecting the stability of international relations [4]. Thus, a new water resources cooperation mechanism is urgently needed to solve these conflicts in the Lancang-Mekong River Basin and promote harmonious and stable relations among countries in the basin.

Game Theory (GT) is the study of mathematical models of conflict and cooperation between intelligent, rational decision-makers. The competition and conflict between the users of public water resources make the application of GT possible. Nash Equilibrium (NE) can be interpreted as the optimal countermeasures of participants against each other. The strategy taken by a given opponent in the optimal strategy is the optimal response to the opponent’s strategy. However, the opponent’s optimal strategy is essentially unknown. Generally, the NE cannot be reached by a simple optimization method. Therefore, the solution of NE is still a significant problem in GT [5]. Meta-heuristic Algorithms (MHAs) can provide a new idea for solving NE, including Remora Optimization Algorithm (ROA), African Vulture Optimization Algorithm (AVOA), Gorilla Troop Optimization algorithm, Genetic Algorithm (GA), and Particle Swarm Optimization (PSO). The evolving technologies based on PSO can simulate the attributes of intelligent selection behavior of biological groups, including the social characteristics of cooperation and learning between organisms, and implement a highly purposeful random search. Thus, PSO has higher computational efficiency and accuracy than other MHAs, more suitable than this paper.

Therefore, PSO is used to solve the NE problem and build a cooperative game model. Firstly, the basic theory of the PSO algorithm and cooperative game model is described. Secondly, the cooperative game model based on the PSO algorithm is described. Finally, the performance of the proposed model is tested through comparison with the GA. Then, this paper takes countries along the Lancang Mekong River Basin as the research object to discuss the water consumption and economic benefits of each state under different cooperation modes. Finally, it puts forward a series of improvement measures and suggestions. The present work aims to provide essential technical support to solve the possible contradictions and conflicts among countries in cross-border watersheds and build harmonious international relations.

## Related work

Climate change (CC) and the response of river runoff to precipitation changes affect watershed systems and ecological functions in different ways [6, 7]. In the Lancang-Mekong River Basin, CC is the main reason for floods and droughts intensification [8]. Wang et al. (2021) proposed a two-stage NSGA-II and Technique for Order Preference by Similarity to an Ideal Solution framework to provide an emergency water supply plan for decision-makers in Lancang Mekong Basin [9]. Bi and Zhu (2020) used the data of the Lancang-Mekong River area from 2009 to 2018 to evaluate the vulnerability of water resources. They found that the vulnerability of water resources in the Lancang-Mekong River Basin was relatively high in the recent ten years, especially in 2016 [10]. Zhang et al. (2020) believed that the Lancang-Mekong River was a vital irrigation water resource for China and five other Southeast Asian countries. The author assessed the drought risk of the Lancang-Mekong River Basin by combining the hazard and vulnerability assessment of drought. The results proved that countries located in the middle and lower reaches of the Mekong River were more prone to drought, and the planting of sugarcane, rice, and cassava was at high risk of drought [11]. Paik et al. (2020) argued that rising sea levels and increasing salinity invasion threatened rice production in the Mekong Delta region of Vietnam. The results indicated that improved rice varieties had been extensively planted in high salinity areas, resulting from the local environment and locational characteristics [12].

Nguyen et al. (2020) investigated the surface water quality in southwest Vietnam, considering that it might be affected by natural factors and human pollution sources. The results showed that pollution sources, such as agricultural production, residential areas, and industrial manufacturing, might directly affect surface water quality. Moreover, the seasonal variation might affect surface water quality by introducing dilution and runoff effects in the dry and rainy seasons [13]. Yun et al. (2020) believed that the operation of the reservoir might reduce or exacerbate the impact of CC on flood events in the Lancang-Mekong River Basin. The reservoir module was added to the variable infiltration capacity model to simulate the effect of the reservoir on runoff. The results showed that the reservoir operations had a significant impact on the runoff of the Lancang-Mekong River Basin from 2008 to 2016 [14]. Yun et al. (2021) suggested that effective reservoir management could reduce the CC-induced extreme drought and humidity in the Lancang-Mekong River Basin. However, there was still limited knowledge about the evolution of extreme hydrological events from CC and the effectiveness of reservoir regulation in the Lancang-Mekong River Basin. They filled the knowledge gap by assessing the effectiveness of reservoir regulation on extreme hydrological changes in the 21st century [15]. Yu et al. (2019) proposed a method to analyze the impact of cascade reservoir system operation on cross-border cooperation under different hydrological conditions through a case study of the Lancang-Mekong River. The economic benefits under cooperation among Lancang-Mekong River Basin countries were more remarkable than a single-country operation, particularly in dry years [16]. Zhu et al. (2021) proposed that establishing a water market in cross-water areas could improve the efficiency of water RU and produce substantial economic value [17].

Liu et al. (2020) constructed a fuzzy alliance game model for transnational water resources allocation in multinational river basins. The research uncovered that compared with the initial allocation strategy based on agricultural water demand, the cooperative approach increased the water resource allocation of high-utilization countries and improved the overall water use rate of the whole basin. Therefore, the fuzzy alliance model was suitable for other multi-ethnic rivers’ water resources allocation [18]. Noori et al. (2020) applied the non-cooperative GT of stability definition to model the bilateral agreements of industrial and agricultural sectors under inequality and studied the allocation of water resources through various stability definitions. The effectiveness of the model was proved by example analysis [19]. Jia et al. (2021) proposed a ROA based on bionics, natural heuristic, and metaheuristic. ROA was mainly inspired by the parasitic behavior of remora [20]. Abdollahzadeh et al. (2021) constructed a new MHA based on the lifestyle of African vultures, namely the AVOA, which simulated the foraging and navigation behavior of African vultures [21].

## Basic theory, algorithm, and concept definition

### PSO algorithm

In nature, birds and fish have the same consistent pattern in some group behaviors. For example, when the group turns suddenly, individuals will not collide. This phenomenon has been widely studied and reconstructed (modeled) through modern computer technologies [22]. The model supposes an optimal distance among individuals in the swarm [23]. Until the end of the 20th century, after the emergence of the PSO algorithm, the individuals in the swarm were considered particles connected through a unique cooperative mechanism, ignoring the volume and mass of particles. The motion state of each particle is affected by the historical motion states of the particle itself and the swarm. Hence, the motion of the particle swarm becomes finding the optimal solution in space [24–26]. In the PSO algorithm, each particle in the group has its own motion state. The position change of a single particle can be expressed by Eq (1).


$$v_{i}^{d}=v_{i}^{d}+c_{1}rand_{1}^{d}(pBest_{i}^{d}-x_{i}^{d})+c_{2}rand_{2}^{d}(gBest^{d}-x_{i}^{d})$$ (1)

In Eq (1), *x*_*i*_ represents the current position of the *i*-th particle; *v*_*i*_ indicates the current velocity of the *i*-th particle; *pBest*_*i*_ denotes the position of the optimal solution sought by the *i*-th particle; *gBest* stands for the position of the historical optimal solution of the whole swarm; *d* signifies the dimension; $rand_{1}^{d}\in [0,1]$, and $rand_{2}^{d}\in [0,1]$; *c*_1_ and *c*_2_ represent learning factors. Eq (2) indicates the velocity variation of a single particle.


$$x_{i}^{d}=x_{i}^{d}+v_{i}^{d}$$ (2)

In Eq (2), *x*_*i*_ represents the current position of the *i*-th particle, *v*_*i*_ denotes the current velocity of the *i*-th particle, and *d* indicates the dimension.

**Fig 1:**
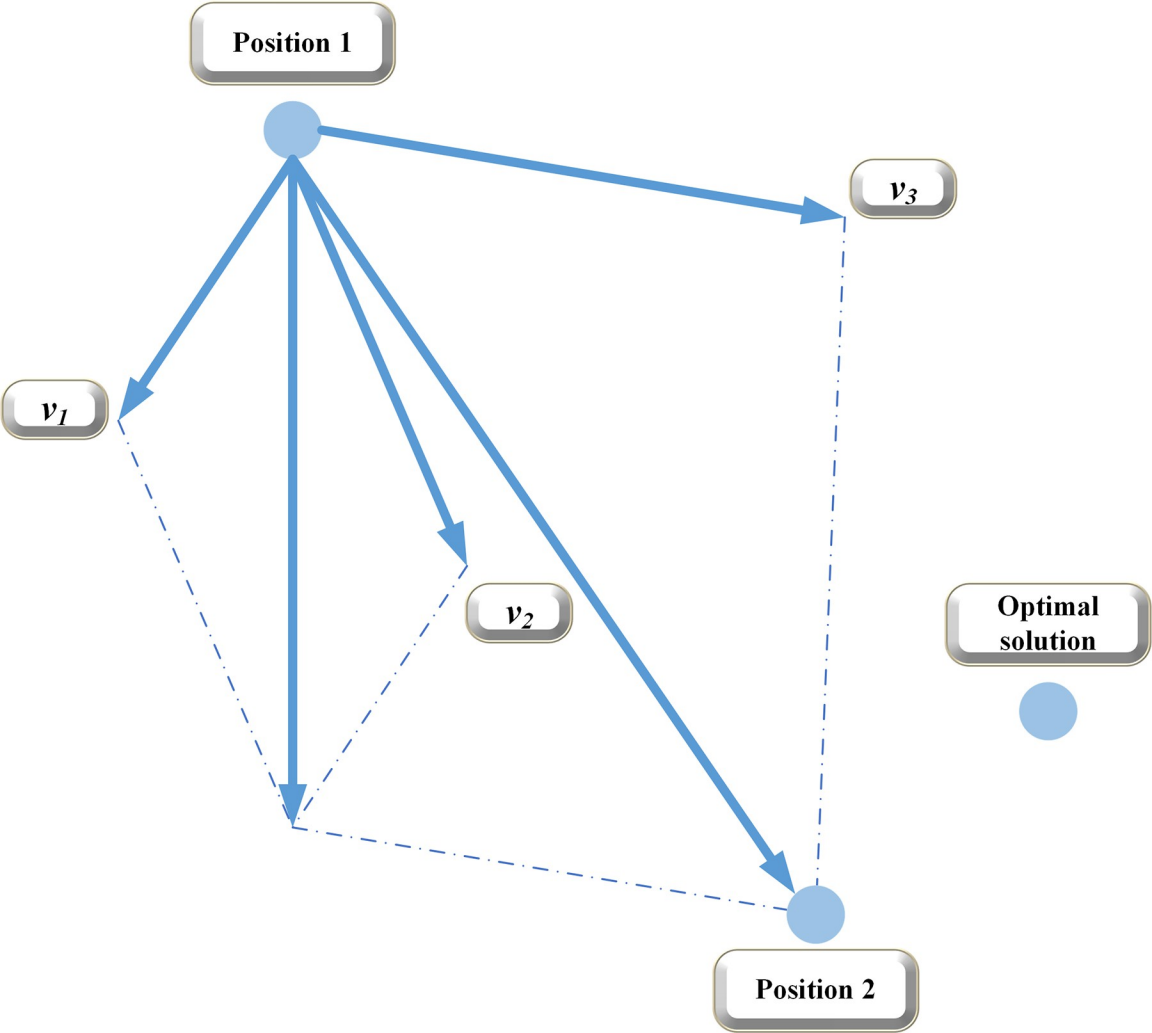
Finding the optimal solution by PSO.

The right side of Eq (1) is composed of particle velocity, "cognition," and "society," which respectively correspond to the influence of the ability to explore new areas, local searchability, and group historical optimal solution. Fig 1 illustrates how the POS algorithm finds the optimal solution in a 2D search space, where *A* is the position of the optimal solution of the swarm.

The *i*-th particle moves from position 1 to position 2, where *v*_*1*_ is the velocity caused by the historical optimal solution of the swarm, *v*_*2*_ represents the velocity caused by the historical optimal solution of the *i*-th particle, and *v*_*3*_ denotes the original speed of the *i*-th particle. The final velocity *v* is the vector synthesis of these three velocities. The above process is repeated until the swarm approaches the position of the optimal solution [27].

The PSO algorithm is simple in structure and fast in convergence, which is suitable for reservoir scheduling. However, the PSO algorithm is prone to the local optimum with fast convergence. Thus, in practical operation, the global search is performed first. When an optimal solution is determined within a specific range, the local search ability is used to search for the optimal solution position [28–30], as shown in Eq (3).


$$v_{i}^{d}=\omega \cdot v_{i}^{d}+c_{1}rand_{1}^{d}(pBest_{i}^{d}-x_{i}^{d})+c_{2}rand_{2}^{d}(gBest^{d}-x_{i}^{d})$$ (3)

**Fig 2:**
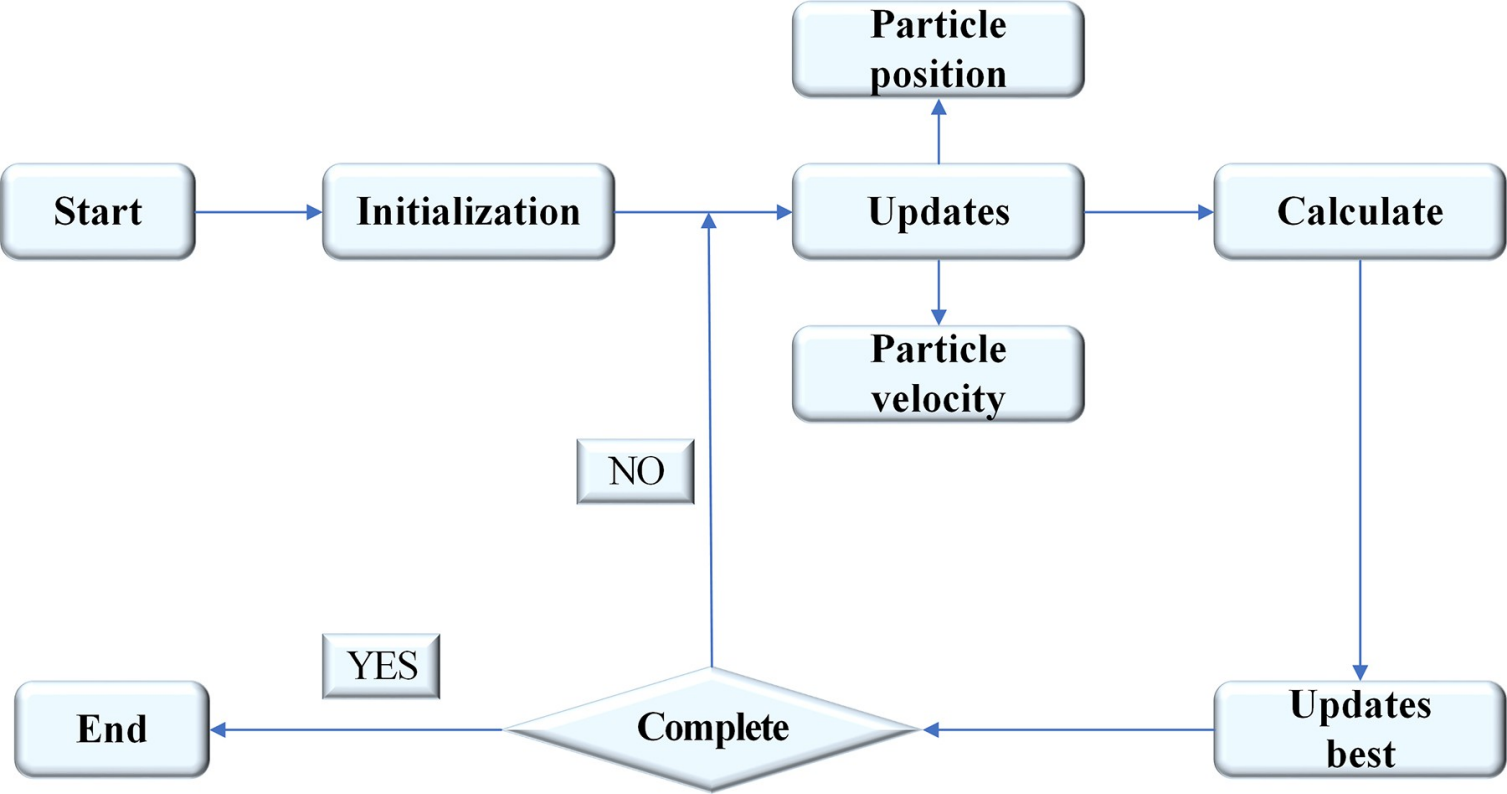
Flow chart of PSO algorithm.

In Eq (3), *ω* represents the inertia weight. The particle velocity on the right side of Eq (1) is multiplied by *ω*. Besides, *ω* is adjusted to avoid the local optimal solution of the PSO algorithm. Generally, the value of the PSO algorithm decreases linearly from 0.9 to 0.4. The PSO algorithm based on Eq (3) is also called the standard PSO algorithm, and its operation flow is shown in Fig 2.

### Cooperative game model

**Fig 3:**
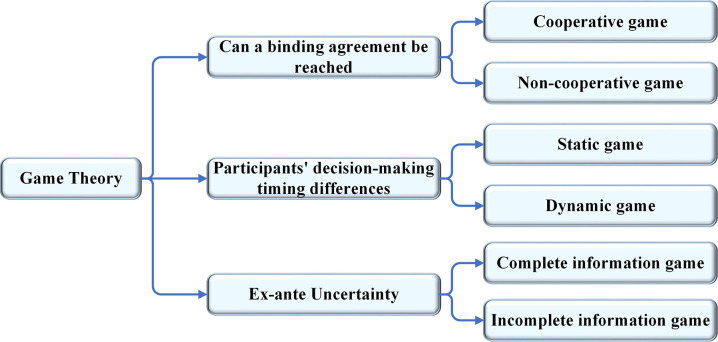
GT classification.

Game Theory (GT) is an analytical means to study the strategic judgment of both participants in an interactive situation [31]. This theory is popular in social and economic fields. With mathematical models, GT shows its good conflict decision-making ability. The use of GT usually involves three parts: participants, actions, and results. Through rational analysis, the profits of the group and the individual are maximized, thus obtaining the optimal solution of the whole group [32], as illuminated in Fig 3.

GT applications include cooperative and non-cooperative games. In a cooperative game, the participants in the group cooperate to maximize the profits of the group and themselves, which is the combination of cooperation and competition. By contrast, a non-cooperative game maximizes the profits of individual participants, which is a pure competitive relationship [33]. Transboundary emergency water supply involves distributing water-related benefits among different stakeholders. The key to solving this problem is to establish a comprehensive model to meet the needs of most stakeholders [34]. This paper studies the benefit distribution of Lancang-Mekong River Basin countries from cooperative GT and realizes the corresponding cooperative game model.

According to the GT, if the number of participants in the game is *N*, Eq (4) exists.


I={1,2,3,…,N} (4)

In Eq (4), the possible game combinations are 2*N*-1 except empty sets. When participants pursue the maximization of their respective profits without cooperation, Eq (5) is satisfied.


v(S)={v(1),v(2),v(3),…,v(N)} (5)

In Eq (5), *S* indicates the collection of participants, and *S*⊂*I*. Besides, *v*(*S*) shows that the participants work alone, so *v*(∅) = 0.


$$v(S_{1}\cup S_{2})\geq v(S_{1})+v(S_{2})$$ (6)

In Eq (6), *S*_1_⊆*I*, *S*_2_⊆*I*, and *S*_1_∩*S*_2_≠∅.


$$v(I)\geq \sum_{i=1}^{N}v(i)$$ (7)

In Eq (7), *v* represents the game characteristic function, [*I*,*v*] is called the *N*-person cooperative game.

Here, the Shapley value method solves the above cooperative game model, as presented in Eq (8).


$$\psi_{i}(v)\geq v(i)$$ (8)

In Eq (8), *i*∈*N*, and *ψ*_*i*_(*v*) represents the profit obtained by the participants.


$$\sum_{i\in S}\psi_{i}=v(I)$$ (9)

In Eq (9), $\sum_{i\in S}\psi_{i}$ represents the sum of profits obtained by all participants in the cooperative game.


$$\psi_{i}(v)=\sum_{S\subset I}W(|S|)[v(S)-v(S\backslash i)]$$ (10)

In Eq (10), *i*∈*N*, |*S*| represents the number of cooperative participants in *S*, and *S*\*i* is the subset obtained after the participant *i* is removed from *S*. Besides, *v*(*S*)−*v*(*S*\*i*) indicates the participant’s contribution (*i*) to the profit of *S*.


$$W(|S|)=\frac{(N-|S|)!(|S|-1)!}{N!}$$ (11)

In Eq (11), *N* represents the number of participants in the game, and |*S*| indicates the number of cooperative participants in *S*.

### Establishment of the cooperative game model based on PSO algorithm

The Lancang-Mekong River Basin water resources are principally used for shipping, hydropower development, agricultural irrigation, and fishery breeding. The game behavior of water resources allocation in cross-border basins is analyzed based on the PSO algorithm. The water environment system planning comprises several interrelated, interactive, and comprehensively benefit-integrated subsystems. This highly complex evolution process can be expressed as:

$$C=\sum_{i=1}^{N}C_{i}[1+\sum_{j=1}^{N}\sum_{i=1}^{N}a_{ij}]$$ (12)

In Eq (12), *C* represents the nonlinear adaptive ability of the water environment system; *a*_*ij*_ denotes the nonlinear interaction coefficient between subsystem *i* and subsystem *j* of each component unit of the system; *Ci* indicates a reasonable response corresponding to the overall system when the two subsystems interact (i.e., a stable state solution). Subsystem *i* stands for the sustainable evolution process of t_0_ ~ ∞ with time from an arbitrary starting time.


$$C_{i}=f(x),\;x={\left(B_{i},S_{i},E_{i},T_{d}\right)}^{T}$$ (13)

In Eq (13), *B*_*i*_, *E*_*i*_, and *T*_*d*_ represent the ecological environment, resource status, social status, economic status, and all dynamic factors, respectively. Due to various complex conditions and factors of the subsystem, Eq (14) can be obtained:

$$C_{i}=f(B_{i},S_{i},E_{i},T_{d})$$ (14)

$$\frac{\partial C_{i}}{\partial_{t}}=\frac{\partial_{C}}{\partial_{B_{i}}}\frac{\partial B_{i}}{\partial_{t}}+\frac{\partial_{C_{i}}}{\partial_{S_{i}}}\frac{\partial_{i}}{\partial_{t}}+\frac{\partial_{C_{i}}}{\partial_{E_{i}}}\frac{\partial_{E}}{\partial_{t}}+\frac{\partial T_{d}}{\partial_{t}}$$ (15)

Due to the nonlinearity, continuity, and complexity of the water environment ecosystem, the whole water environment can be regarded as a subsystem based on rules and unit autonomy. It makes full use of information and regeneration ability to guide the optimal direction of the whole development system. During the evolution of the water environment system, the actors involved can change their behavior through learning to achieve mutual coordination and adaptation with the external environment. If there are k populations in the system, each population k (0, 1, 2, 3,…, n) has N strategies, then the N-dimensional vector set corresponding to population k is expressed as in Eq (16):

$$s^{k}=\{X=(X_{1},X_{2},\cdots ,X_{N})X_{I}⩾0),\;X1+X2+\ldots +XN=1\}$$ (16)

Any vector *r*^*k*^ in this form is the hybrid strategy of any individual of population *K*. The vector in this form represents the proportion of individuals adopting each approach in population *K*. Evolutionary GT uses the fitness of individuals in each population as the payoff to describe the game strategy. The fitness of individuals is a function of individual schemes and current states. The fitness function is recorded as

$$f(r,s)=\{f^{1}(r^{1},s),f(r^{2},s),\cdots ,f(r^{k},s)\}$$ (17)

The fitness function calculates the average fitness to obtain the strategy growth rate. Assuming that the strategy growth rate is equal to its relative adaptability, as long as the fitness of a strategy is higher than the average fitness of the group, the strategy will develop. The objective function of the benefit evaluation model of water resources supply in cross-border basins reads:

$$\max\;Z=\max\left\{\underset{t}{\sum }\overset{\underset{k}{i=1}}{\sum }(p_{i}^{e}\times x_{i}^{e}+p_{i}^{a}\times x_{i}^{a}+p_{i}^{f}\times x_{i}^{f}+p_{i}^{s}\times x_{i}^{s}+Ph_{i,t})\right\}$$ (18)

**Fig 4:**
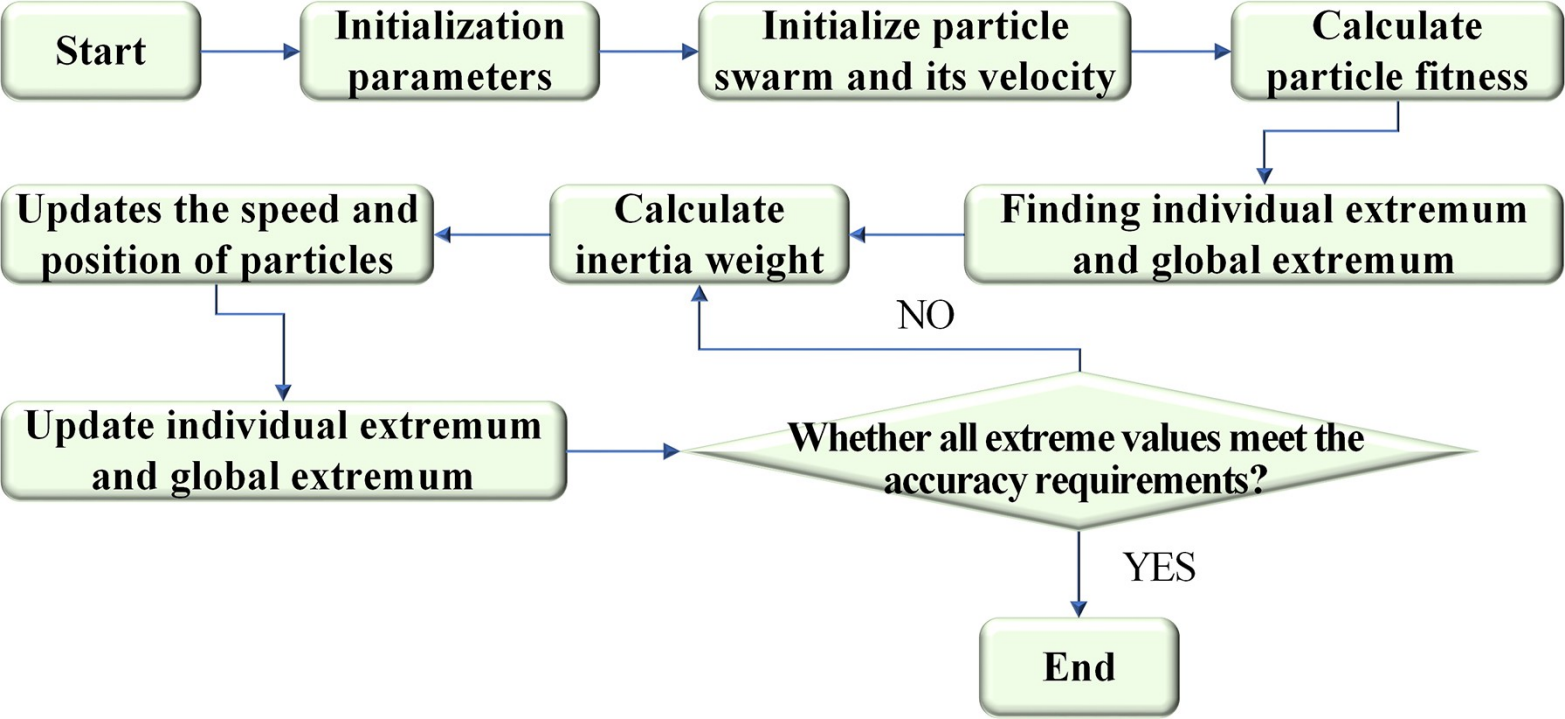
Algorithm flow.

In other words, on the premise of meeting various constraints, the maximum benefits of power generation, agriculture, fishery, shipping, and disaster prevention in the whole basin can be sought. In Eq (18), *Ph*_*i*,*t*_ denotes disaster prevention benefits; *t* represents different periods, including ordinary years, extreme drought, catastrophic floods, and emergencies; *K* stands for the river diversion section, referring to the diversion section between countries; *Z* indicates the water benefit of the whole basin. $p_{i}^{e}$, $x_{i}^{e}$, $p_{i}^{a}$, $x_{i}^{a}$, $p_{i}^{f}$, $x_{i}^{f}$, $p_{i}^{s}$, and $x_{i}^{s}$ are the benefits of unit water consumption for power generation, water consumption for power generation, benefit per unit of agricultural water consumption, agricultural water consumption, benefits of unit water consumption for the fishery, water consumption for the fishery, benefits of unit water consumption of shipping water, water consumption for shipping, respectively. Agricultural water consumption is consumption water, and the rest are guaranteed water. Fig 4 reveals the workflow of the cooperative game model based on the PSO algorithm.

### Case study

#### (1) Overview of the Lancang-Mekong River

**Fig 5:**
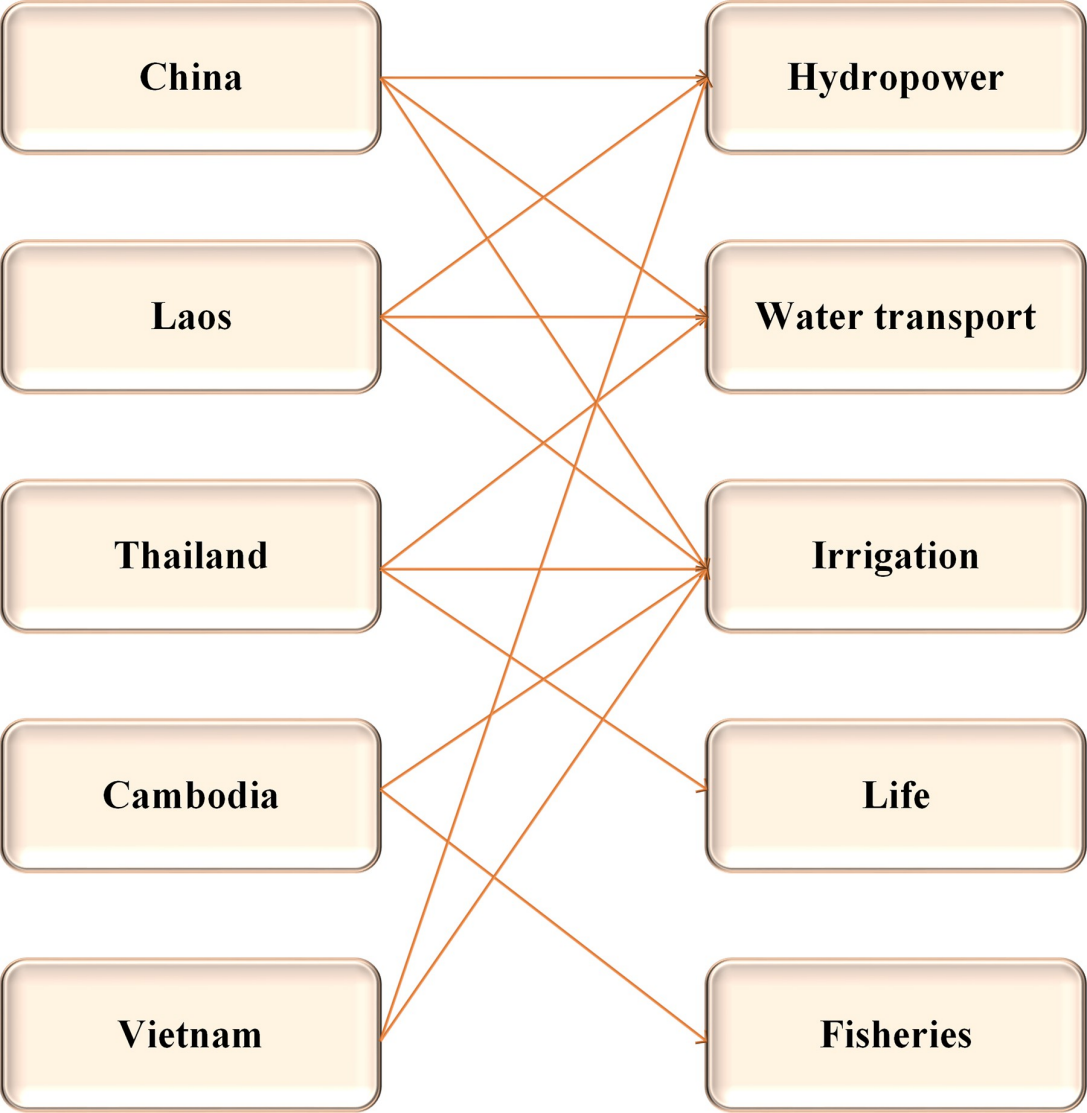
Comparison of GDP or water use income among basin countries.

Lancang-Mekong River originates in Qinghai Province, China. It is called the Lancang River in China and the Mekong River outside China. The Lancang-Mekong River Basin has extremely great water RU potential. Among the six countries sharing the River Basin (China, Myanmar, Laos, Thailand, Cambodia, and Vietnam), Myanmar accounts for a tiny watershed area and yield, so Myanmar is not considered in the cooperative game here [35]. In the Lancang-Mekong River Basin, the water resources profits of various countries are mainly concentrated in irrigation, power generation, and water transportation, as shown in Fig 5.

#### (2) Data source and calculation

The sample data are selected from the Water Conservancy Statistical Yearbook 2020 by China’s Ministry of Water Resources in 2020. Therefore, the data is open to the public. In 2016, China implemented emergency water replenishment for Myanmar, Thailand, Laos, Cambodia, and Vietnam according to the Lancang-Mekong cooperation mechanism out of cooperative sincerity, which has gained both praise and criticism from the five countries [36, 37]. Undeniably, water resources cooperation concerns the vital profits of the countries along the basin. Here, the irrigation water resources allocation is mainly analyzed for the Lancang-Mekong River Basin countries. Myanmar, which only occupies a small part of the Lancang-Mekong River Basin and has little impact on the irrigation water of the other basin countries, is not considered. The proportional method determines the water resource allocation, as shown in Eq (19).


$$Q_{j}=A_{j}\times Q_{I}$$ (19)

In Eq (19), *Q*_*j*_ represents the amount of water distributed by *j* Country, *A*_*j*_ denotes the water utilization rights distributed by the proportion for *j* Country, and *Q*_*I*_ indicates the total amount of irrigation water for the five countries, which is 130 billion cubic meters.


$$A_{j}=\frac{S_{j}}{\sum_{i=1}^{5}S_{I}}$$ (20)

In Eq (20), *S*_*j*_ is the area of food security production in *j* country, and $\sum_{i=1}^{5}S_{I}$ represents the sum of the areas of food security production in the five countries.

The watershed area-based division method linearly calculates the total amount of water resources and each country’s proportion of the watershed area in the entire watershed area of the cross-border river. Suppose *Q*, *A*, *r*, and *Q*_*i*_ represent the total amount of water resources in the basin, the total watershed area, the watershed area within each basin country, and the water volume obtained by each basin country, respectively. In that case, Eq (21) is obtained:

$$Qi=Q\times \frac{r}{A}$$ (21)

The average annual runoff of the Lancang-Meigong river basin is 415 billion cubic meters, with a total watershed area of 811,000 square kilometers. The watershed areas of China, Laos, Thailand, Cambodia, and Vietnam are 167,000 square kilometers, 215,000 square kilometers, 182,000 square kilometers, 161,000 square kilometers, and 65,000 square kilometers, respectively. Each country’s proportion of the watershed area can be calculated.

#### (3) Algorithm parameter setting and running environment

This paper mainly draws lessons from the parameter setting of the algorithm in Jane (2021) [38]: the particle swarm size is 10, the maximum number of iterations is 1,000, the maximum inertia weight is 0.9, the minimum inertia weight is 0.4, and the accuracy requirement is 10–5. This model runs on the hardware platform of Windows 10 Operation System, 3.40GHz Central Processing Unit (CPU), and 8G Memory. Additionally, the algorithm is implemented by Matlab 7.0 computer program language.

## Benefit analysis results of water resources allocation in the Lancang-Mekong River Basin countries

### Results of model performance test

**Fig 6:**
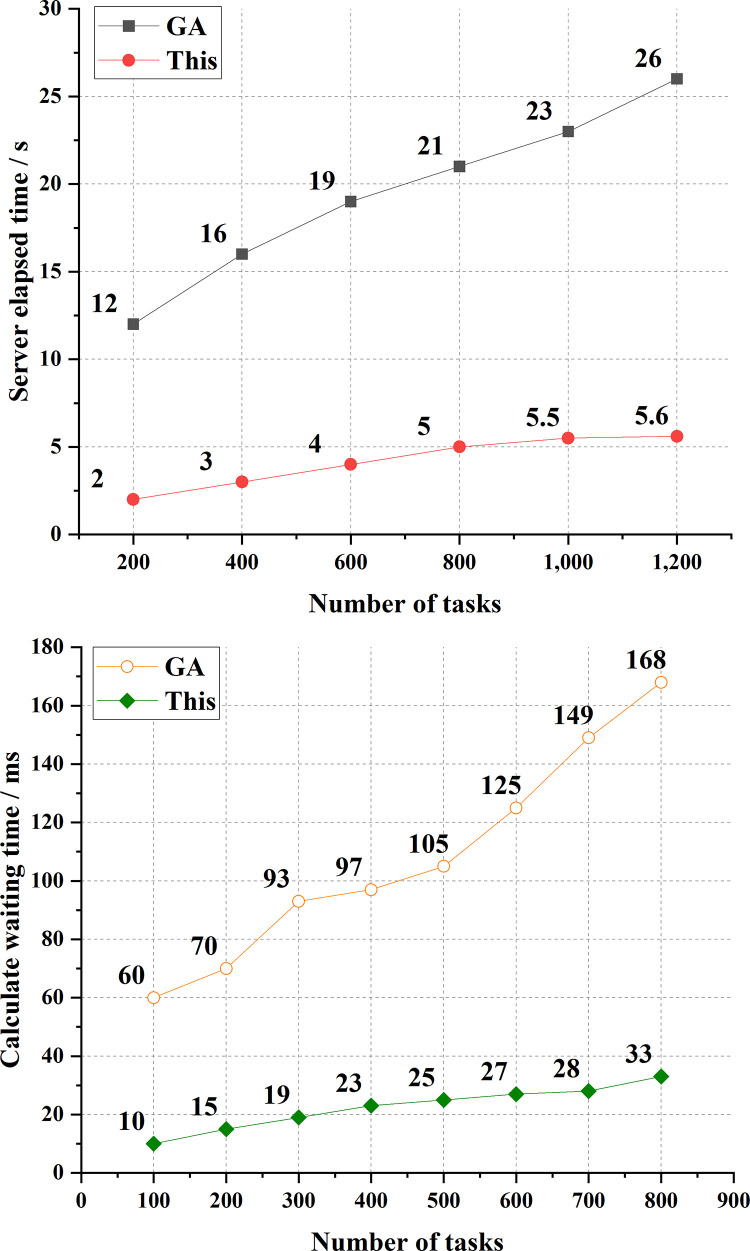
Model performance test results (a. Server occupancy time; b. Calculation waiting time).

Subsequently, this paper takes the server occupation time and waiting time during calculation as the test indexes and compares the results with the GA to verify the performance of the proposed PSO-based cooperative game model. The test results are plotted in Fig 6.

As plotted in Fig 6, GA’s average server occupation time and the proposed algorithm under different task quantities are 19.5s and 4.2s, respectively. Thus, the proposed algorithm beats the GA by 78.46%. Besides, the average calculation waiting time of GA and the proposed algorithm under different tasks is 108.4ms and 22.5ms, respectively. Hence, the proposed algorithm is 79.24% lower than the GA. In short, the proposed PSO-based cooperative game model has higher computational efficiency and excellent performance than the GA and is more suitable for the current research.

### Water resources allocation

**Fig 7:**
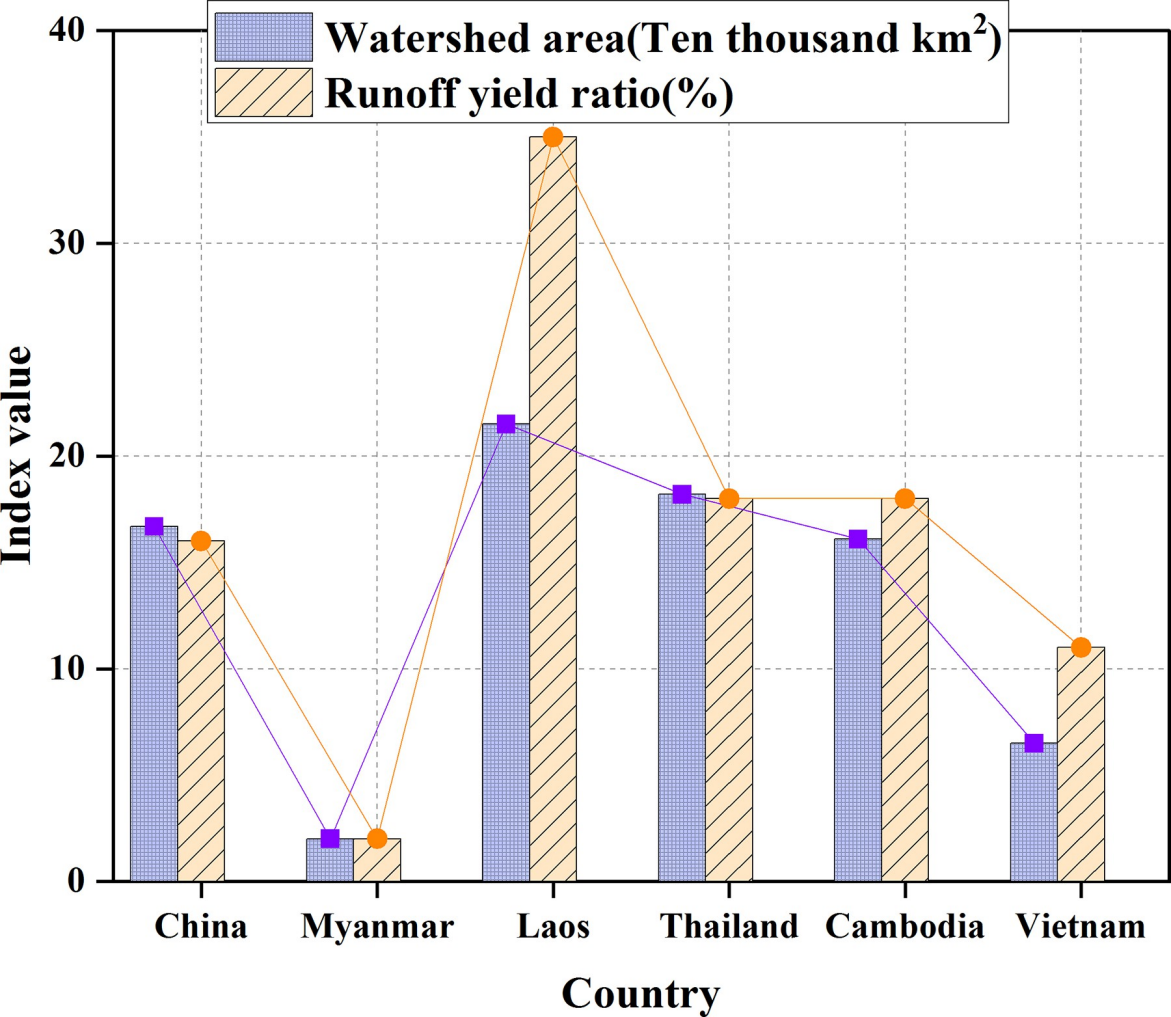
Comparison of watershed area and runoff yield ratio in various countries.

Table 1 and Fig 7 show the watershed area of Lancang-Mekong River Basin countries and their corresponding runoff yield ratio.

**Table 1:** Watershed area and corresponding runoff yield ratio of the Lancang-Mekong River Basin countries.

Countries	Watershed area/(10,000/km^2^)	Runoff yield ratio/(%)
China	16.7	16
Myanmar	2	2
Laos	21.5	35
Thailand	18.2	18
Cambodia	16.1	18
Vietnam	6.5	11

**Fig 8:**
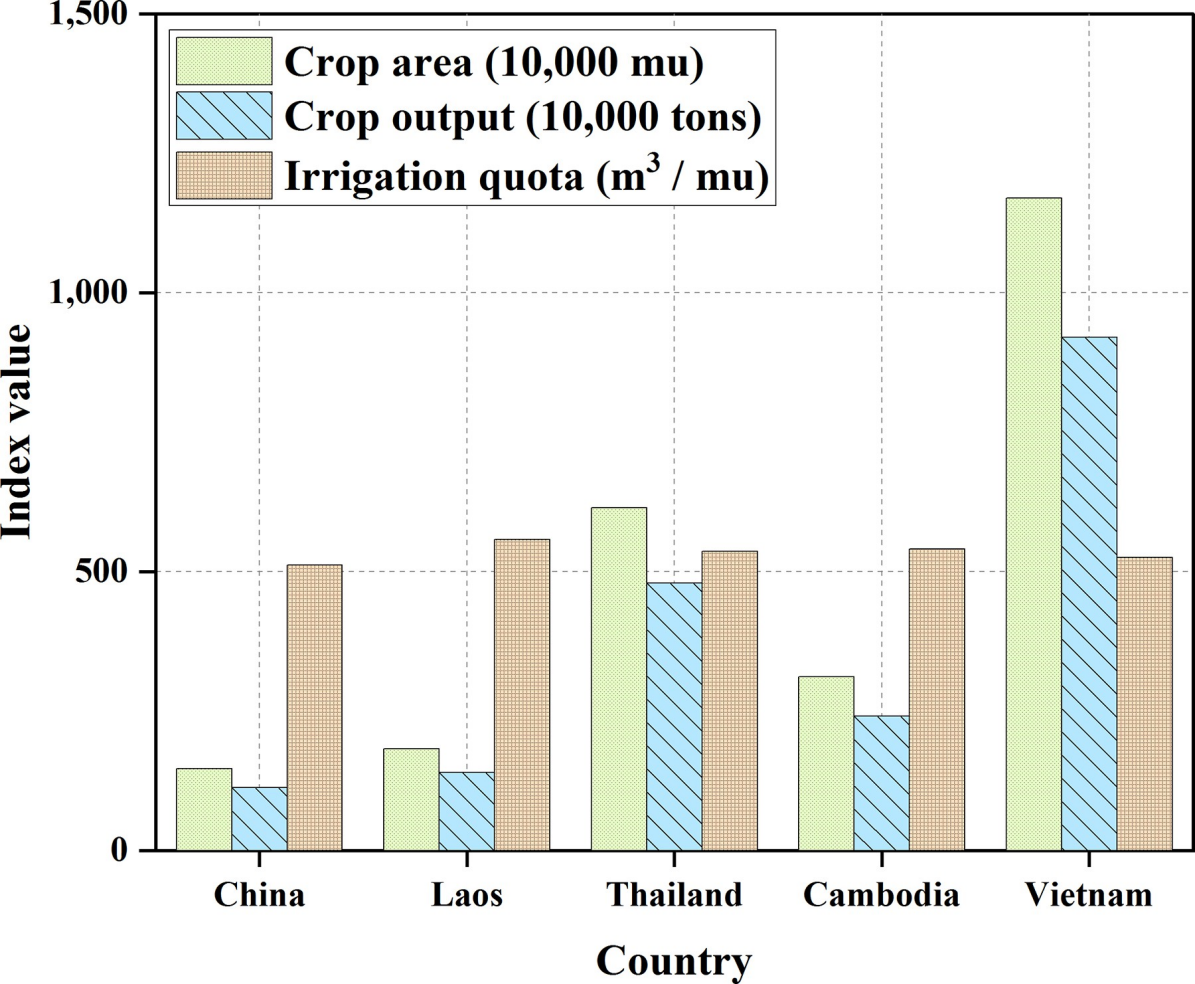
Comparison of crop conditions in various countries.

As shown in Fig 7, the drainage area and runoff yield ratio are arranged from large to small: Laos, Thailand, China, Cambodia, Vietnam, and Myanmar. Since Myanmar accounts for a tiny proportion of the Lancang-Mekong River Basin, the current experiment ignores its contribution. The general crops of the other five countries are shown in Table 2 and Fig 8.

**Table 2:** Crop status of five countries in the Lancang-Mekong River Basin.

Countries	Planting area/(10,000 mu)	Grain production/(10,000 tons)	Irrigation quota/(cm^3^/mu)
China	146.97	113.46	512
Laos	182.77	140	558
Thailand	614.6	480	537
Cambodia	311.48	241.4	541
Vietnam	1170	920.6	526

**Fig 9:**
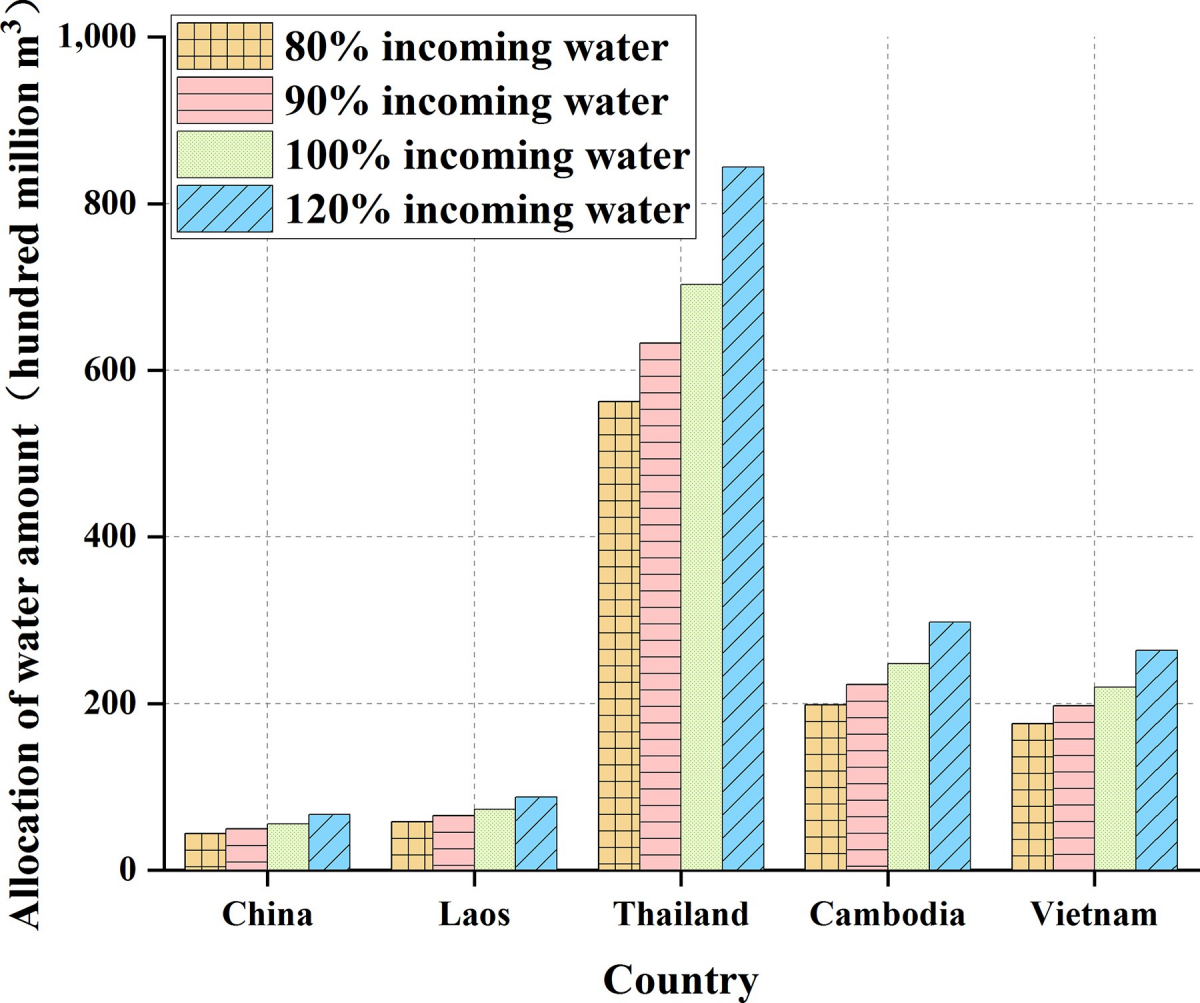
Comparison of water resources allocation in five countries.

According to Fig 8, the Water resources allocation of each country is carried out according to the proportional method, and the Water resources allocation of the five countries is shown in Table 3 and Fig 9. According to Eq (21), the amount of water obtained for each country can be calculated as follows:

$$Q_{China}=Q\times \frac{r_{China}}{A}=4750\times \frac{16.7}{81.1}=978.1(Billion\;m^{3})$$ (22)

$$Q_{Laos}=Q\times \frac{r_{Laos}}{A}=4750\times \frac{21.5}{81.1}=1259.2(Billion\;m^{3})$$ (23)

$$Q_{\mathrm{Thailand}}=Q\times \frac{r_{\mathrm{Thailand}}}{A}=4750\times \frac{18.2}{81.1}=1066.0(Billion\;m^{3})$$ (24)

$$Q_{\mathrm{Vietnam}}=Q\times \frac{r_{\mathrm{Vietnam}}}{A}=4750\times \frac{6.5}{81.1}=380.7(Billion\;m^{3})$$ (25)

$$Q_{\mathrm{Cambodia}}=Q\times \frac{r_{\mathrm{Cambodia}}}{A}=4750\times \frac{16.1}{81.1}=943.0(Billion\;m^{3})$$ (26)

**Table 3:** Allocation of water resources in five countries of the Lancang-Mekong River Basin.

Countries	80% water inflow / (100 million cm^3^)	90% water inflow/(100 million cm^3^)	100% water inflow/(100 million cm^3^)	120% water inflow/(100 million cm^3^)
China	44.5	50.1	55.6	66.8
Laos	58.5	65.9	73.2	87.8
Thailand	562.5	632.8	703.1	843.7
Cambodia	198.6	223.4	248.2	297.8
Vietnam	175.9	197.9	219.9	263.8

**Fig 10:**
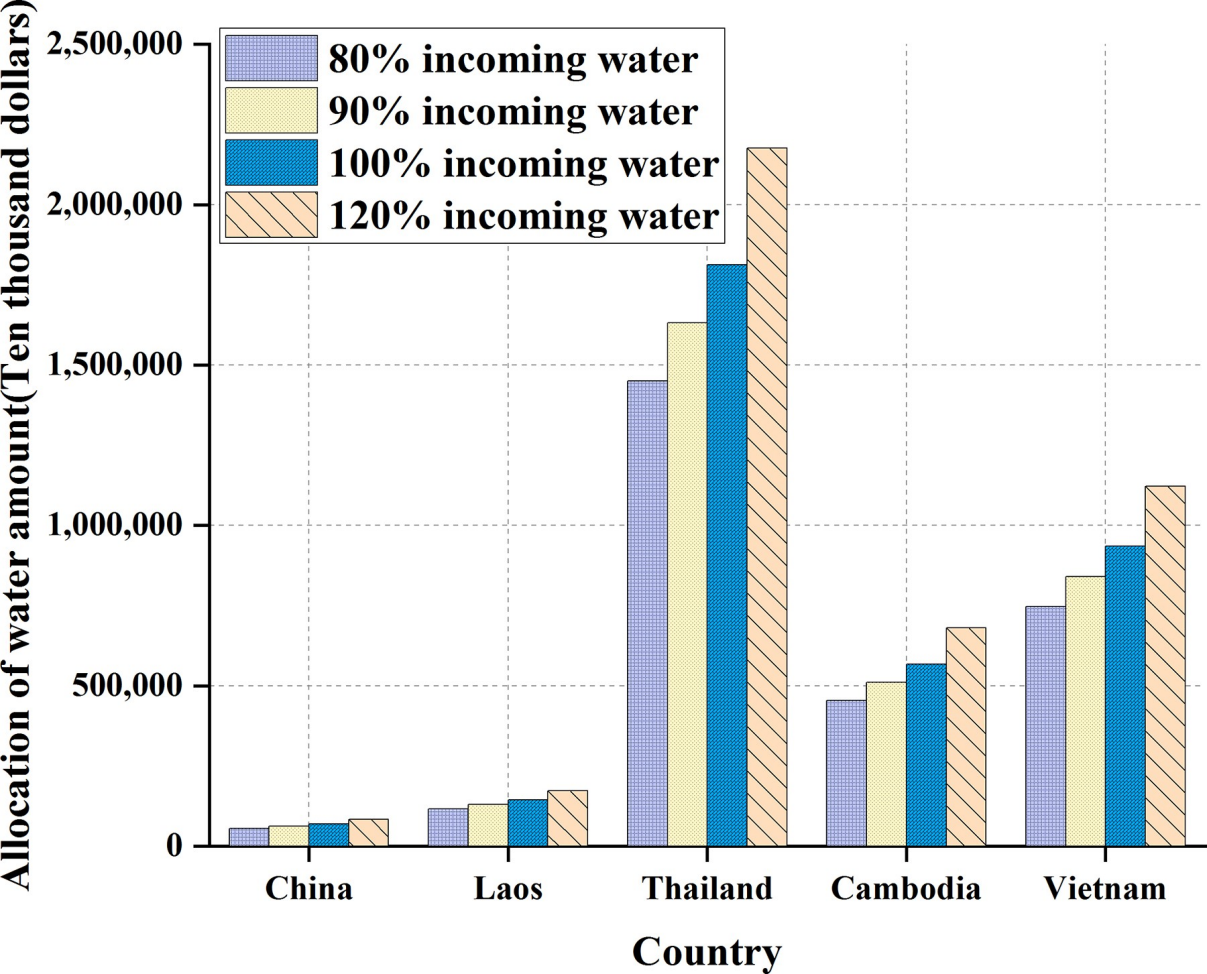
Comparison of grain production benefits under the water resources allocation in five countries.

Fig 9 suggests the total water inflow is also different due to different watershed areas in various countries. Among them, Thailand has the highest total water inflow, followed by Cambodia and Vietnam, and China and Laos have lower water inflow. The grain production benefits of the five countries are shown in Table 4 and Fig 10.

**Table 4:** Benefits of grain production under the allocation of water resources in five countries.

Countries	80% water inflow/(USD 10,000)	90% water inflow/(USD 10,000)	100% water inflow/(USD 10,000)	120% water inflow/(USD 10,000)
China	55,667	62,626	69,584	83,501
Laos	115,726	130,192	144,658	173,589
Thailand	1,450,451	1,631,758	1,813,064	2,175,677
Cambodia	453,983	510,731	567,479	680,975
Vietnam	747,965	841,460	934,956	1,121,947

As shown in Fig 10, the grain production benefits of the five countries are positively correlated with their water resources allocation. Thailand has the highest total water inflow, so its food production efficiency is also the highest, followed by Vietnam and Cambodia, and finally Laos and China.

### Results of Chinese benefits

**Fig 11:**
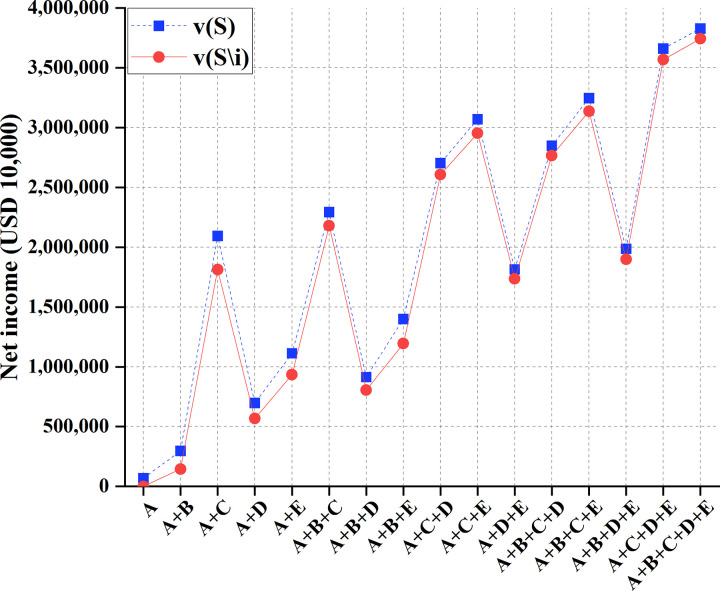
Profit comparison of 16 cooperation modes among China and other four countries.

To sum up, Myanmar has the most negligible impact. China, Laos, Thailand, Cambodia, and Vietnam are represented as *A*, *B*, *C*, *D*, and *E*. Meanwhile, countries must consider the whole basin to maximize their overall interests. They should do comprehensive planning within this overall framework, make rational use of various resources in the basin, integrate and utilize multiple resources reasonably and effectively, and maximize RU benefits. Additionally, from the perspective of environmental protection and development, the society and economy of the basin will show good development only if the water environment of the basin is kept hygienic and healthy. Given water environment problems, such as drought, flood, and severe pollution, all countries in the river basin should actively take adequate measures to improve the water environment in the river basin. And there should be suitable measures and planning so that the actions of each country can form an effective linkage mechanism to minimize the disaster of water pollution and maximize the interests of countries in the basin. Overall, there are 16 cooperation modes between China and the other four countries, namely, *S* = {(A), (A,B), (A,C), (A,D), (A,E), (A,B,C), (A,B,D), (A,B,E), (A,C,D), (A,C,E), (A,D,E), (A,B,C,D), (A,B,C,*E*), (A,B,D,*E*), (A,C,D,E), (A,B,C,D,E)}. Then, *v*(*S*) and *v*(*S*\*i*) are calculated, representing the profit of the 16 cooperation modes and the profit after stakeholder *i* = *A* is removed, respectively. The results are shown in Table 5 and Fig 11.

**Table 5:** Profits of 16 cooperation modes among China and other four countries.

Cooperation mode	*v*(*S*)/(USD 10,000)	*v*(*S\i*)/(USD 10,000)
A	69,584	0
A+B	296,966	144,658
A+C	2,093,568	1,813,064
A+D	696,973	567,479
A+E	1,112,680	934,956
A+B+C	2,293,126	2,179,437
A+B+D	913,769	805,643
A+B+E	1,398,579	1,193,578
A+C+D	2,702,842	2,607,431
A+C+E	3,069,63	2,953,422
A+D+E	1,813,929	1,734,619
A+B+C+D	2,847,582	2,766,584
A+B+C+E	3,245,718	3,136,550
A+B+D+E	1,986,892	1,898,346
A+C+D+E	3,660,724	3,568,579
A+B+C+D+E	3,827,491	3,743,159

When the number of participants in the transaction increases, the benefit from other cross-border water resources development, utilization, and allocation methods will increase in any case of water inflow.

**Fig 12:**
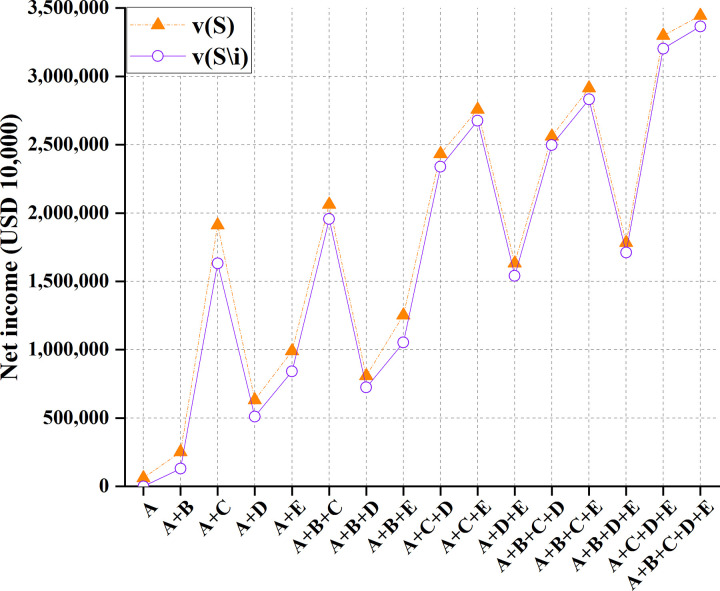
Comparison of profits between China and other countries under 90% water inflow.

Under a pessimistic situation with a 90% water inflow, the benefits are calculated for the cooperation among China and other countries and after removing relevant stakeholders, as shown in Table 6 and Fig 12.

**Table 6:** Profits of China and other countries at 90% water inflow.

Cooperation mode	*v*(*S*)/(USD 10,000)	*v*(*S\i*)/(USD 10,000)
A	62,626	0
A+B	251,253	130,192
A+C	1,912,635	1,631,758
A+D	632,386	510,731
A+E	990,237	841,460
A+B+C	2,061,525	1,956,184
A+B+D	809,128	723,727
A+B+E	1,251,368	1,052,688
A+C+D	2,432,535	2,338,298
A+C+E	2,756,795	2,675,631
A+D+E	1,632,869	1,540,877
A+B+C+D	2,561,689	2,498,126
A+B+C+E	2,913,877	2,831,605
A+B+D+E	1,782,684	1,711,065
A+C+D+E	3,297,562	3,202,576
A+B+C+D+E	3,445,043	3,365,395

Figs 11 and 12 suggest that due to the sharing of water areas in the Lancang-Mekong River Basin, the cooperative game can distribute water rights in the basin. By calculating the benefit distribution of the cooperative game, the total benefits can be fairly distributed among partners. The "core" of game analysis solves the concept of multiple solutions. Thereupon, single-point solutions can be solved. In the year of regular water inflow, there is little difference between the two distribution effects; in the wet years of the basin, the side effects of the two-part allocation method are minor and are not easy to cause floods. Under the same number of participants, the total benefits obtained by the game are different. Therefore, each country can choose the trading partner carefully.

### Results of the profits of remaining four countries

**Fig 13:**
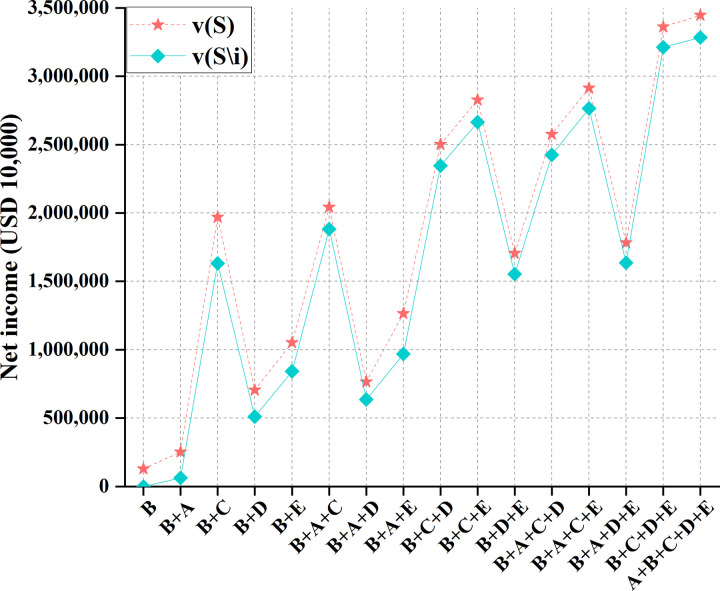
Comparison of profits of 16 cooperation modes between Laos and other four countries.

Similarly, the cooperation profits are calculated for the other four countries: Laos, Thailand, Cambodia, and Vietnam, under a pessimistic situation with a water inflow of 90%. As a result, the comparison between the benefits of cooperation between Laos and other countries and the benefits after removing relevant stakeholders is shown in Table 7 and Fig 13.

**Table 7:** Profits of 16 cooperation modes between Laos and other four countries.

Cooperation mode	*v*(*S*)/ (USD 10,000)	*v*(*S\i*) (USD 10,000)
B	130,192	0
B+A	252,492	62,626
B+C	1,968,436	1,631,758
B+D	705,137	510,731
B+E	1,053,051	841,460
B+A+C	2,042,948	1,882,765
B+A+D	765,144	635,944
B+A+E	1,265,093	968,391
B+C+D	2,501,943	2,346,294
B+C+E	2,826,184	2,664,350
B+D+E	1,705,761	1,552,464
B+A+C+D	2,576,155	2,425,684
B+A+C+E	2,913,496	2,764,964
B+A+D+E	1,783,065	1,634,962
B+C+D+E	3,361,504	3,211,694
A+B+C+D+E	3,446,453	3,284,271

**Fig 14:**
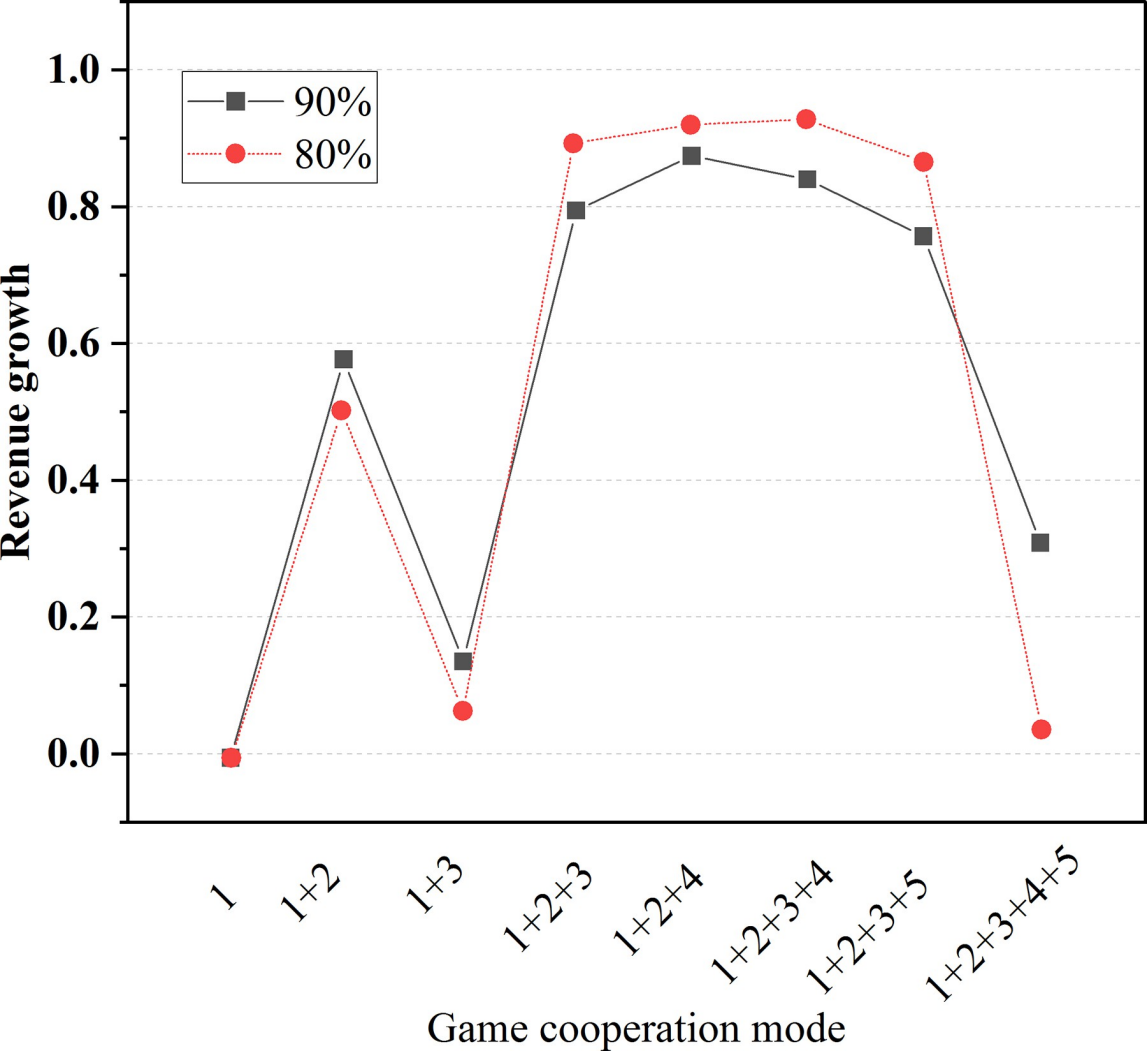
Trend of total benefit under different game combinations (China).

The benefit growth of the two allocation methods in China and other countries under different game combinations and water inflow is shown in Table 8 and Fig 14.

**Table 8:** Benefit growth of two allocation modes in China and other countries under different game combinations and water inflow.

Game combination	90%	80%
1	-0.00604	-0.00606
1+2	0.57662	0.50156
1+3	0.13508	0.0623
1+2+3	0.79441	0.8926
1+2+4	0.87419	0.91977
1+2+3+4	0.83998	0.92765
1+2+3+5	0.75666	0.86539
1+2+3+4+5	0.3086	0.03504

In Fig 14, 1: means China; 2: stands for Laos; 3: represents Thailand; 4: indicates Cambodia; 5: denotes Vietnam. Obviously, when the number of countries participating in the game is within 3–4, the greater the benefit growth is, the benefits of each country are significantly higher than that of utilizing water resources alone under the mode of cooperation.

**Fig 15:**
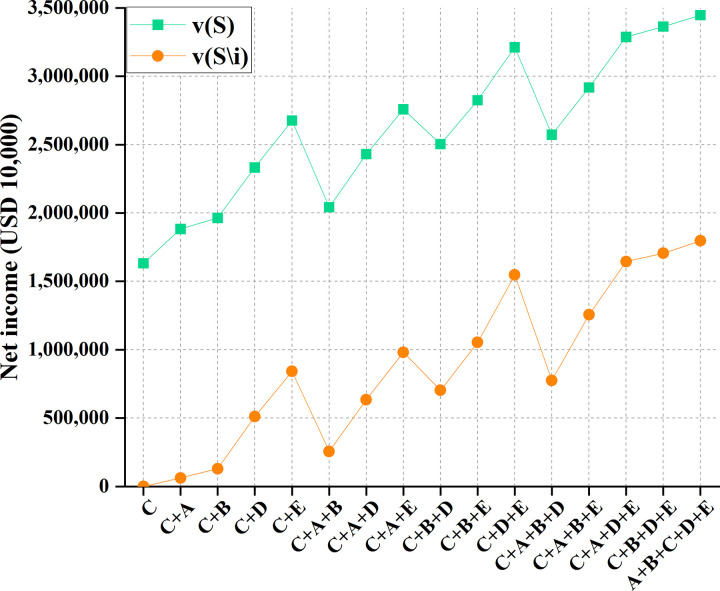
Profit comparison of 16 cooperation modes between Thailand and other four countries.

Under 90% water inflow, the comparison between the benefits of cooperation between Thailand and other countries and the benefits after removing relevant stakeholders is shown in Table 9 and Fig 15.

**Table 9:** Profits of 16 cooperation modes between Thailand and other four countries.

Cooperation mode	*v*(*S*)/ (USD 10,000)	*v*(*S\i*)/(USD 10,000)
C	1,631,758	0
C+A	1,882,454	62,626
C+B	1,963,796	130,192
C+D	2,332,598	510,731
C+E	2,675,985	841,460
C+A+B	2,042,498	256,343
C+A+D	2,430,268	634,935
C+A+E	2,758,461	981,350
C+B+D	2,504,136	702,695
C+B+E	2,824,362	1,053,897
C+D+E	3,211,737	1,546,749
C+A+B+D	2,571,624	775,365
C+A+B+E	2,917,283	1,256,139
C+A+D+E	3,287,133	1,645,014
C+B+D+E	3,362,988	1,704,988
A+B+C+D+E	3,446,453	1,796,484

**Fig 16:**
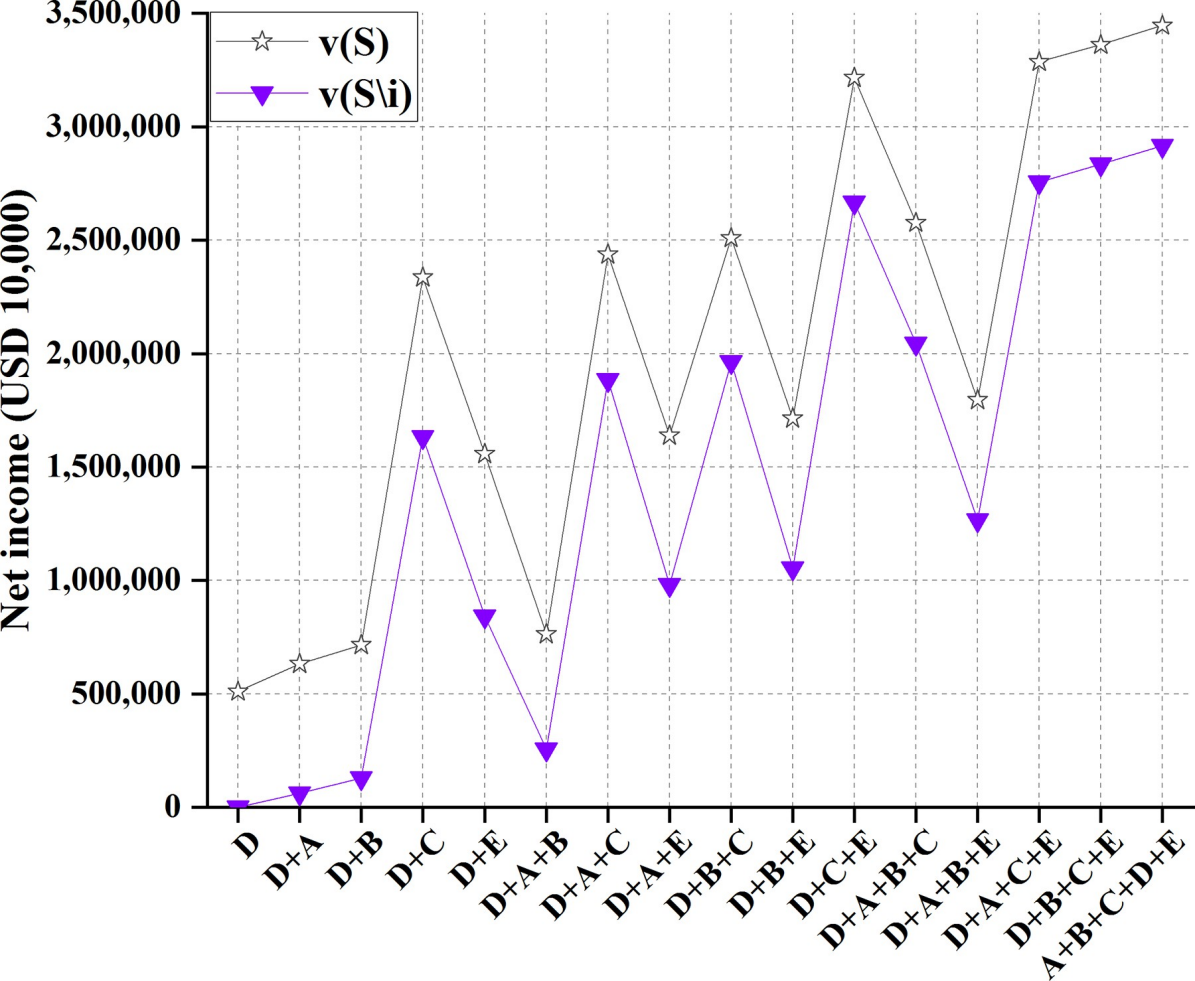
Comparison of profits of 16 cooperation modes between Cambodia and other four countries.

Under 90% water inflow, the comparison between the benefits of cooperation between Cambodia and other countries and the benefits after removing relevant stakeholders is shown in Table 10 and Fig 16.

**Table 10:** Profits of 16 cooperation modes between Cambodia and other four countries.

Cooperation mode	*v*(*S*)/(USD 10,000)	*v*(*S\i*)/(USD 10,000)
D	510,731	0
D+A	632,946	62,626
D+B	715,835	130,192
D+C	2,336,549	1,631,758
D+E	1,557,633	841,460
D+A+B	763,528	256,343
D+A+C	2,436,957	1,884,935
D+A+E	1,639,438	981,350
D+B+C	2,509,826	1,962,695
D+B+E	1,713,897	1,053,897
D+C+E	3,215,964	2,666,749
D+A+B+C	2,576,934	2,045,365
D+A+B+E	1,795,689	1,266,139
D+A+C+E	3,286,154	2,755,014
D+B+C+E	3,361,035	2,834,988
A+B+C+D+E	3,446,453	2,916,484

**Fig 17:**
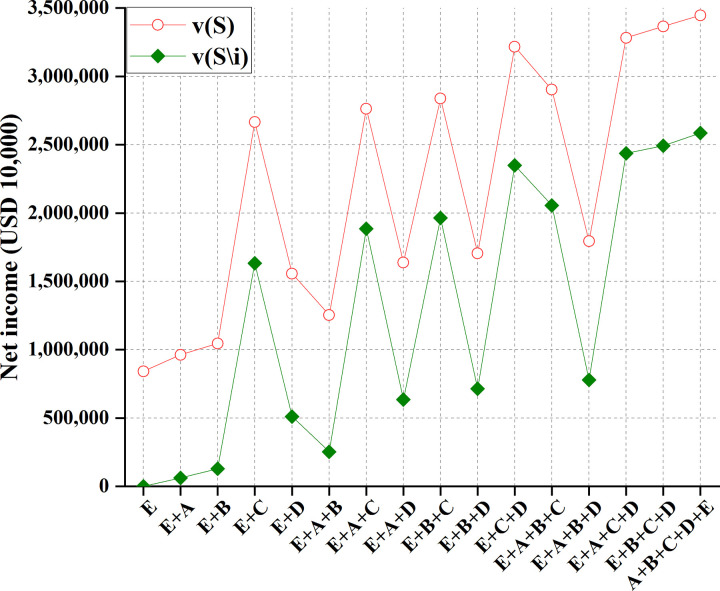
Profit comparison of 16 cooperation modes between Vietnam and other four countries.

Under 90% water inflows, the comparison between the benefits of cooperation between Vietnam and other countries and the benefits after removing relevant stakeholders is shown in Table 11 and Fig 17.

**Table 11:** Profits of 16 cooperation modes between Vietnam and other four countries.

Cooperation mode	*v*(*S*)/(USD 10000)	*v*(*S\i*)/(USD 10,000)
E	841,460	0
E+A	962,535	62,626
E+B	1,045,387	130,192
E+C	2,666,238	1,631,758
E+D	1,556,484	510,731
E+A+B	1,253,730	253,492
E+A+C	2,762,983	1,885,028
E+A+D	1,638,394	634,984
E+B+C	2,838,166	1,964,397
E+B+D	1,704,965	713,847
E+C+D	3,216,895	2,349,283
E+A+B+C	2,902,498	2,056,439
E+A+B+D	1,793,898	778,377
E+A+C+D	3,281,698	2,436,153
E+B+C+D	3,364,984	2,492,837
A+B+C+D+E	3,446,453	2,586,139

Figs 14–17 illustrate that for the remaining four countries, the benefits of developing water resources in the Lancang-Mekong River Basin alone are lower than the net benefits under the five-country cooperation mode, with the maximized benefit under full participation of five countries in the cooperation. Therefore, it is necessary to establish a community of common destiny for all countries in the Lancang-Mekong River Basin. Such a community can reduce the possibility of intervention from international forces and organizations and avoid intensifying contradictions and conflicts of profits along the Basin. All countries in the Lancang-Mekong River Basin can get excellent benefits. Further, China’s "Belt and Road Initiative" strategy will drive the development of countries along the Basin.

### Summary of benefits of five countries

**Fig 18:**
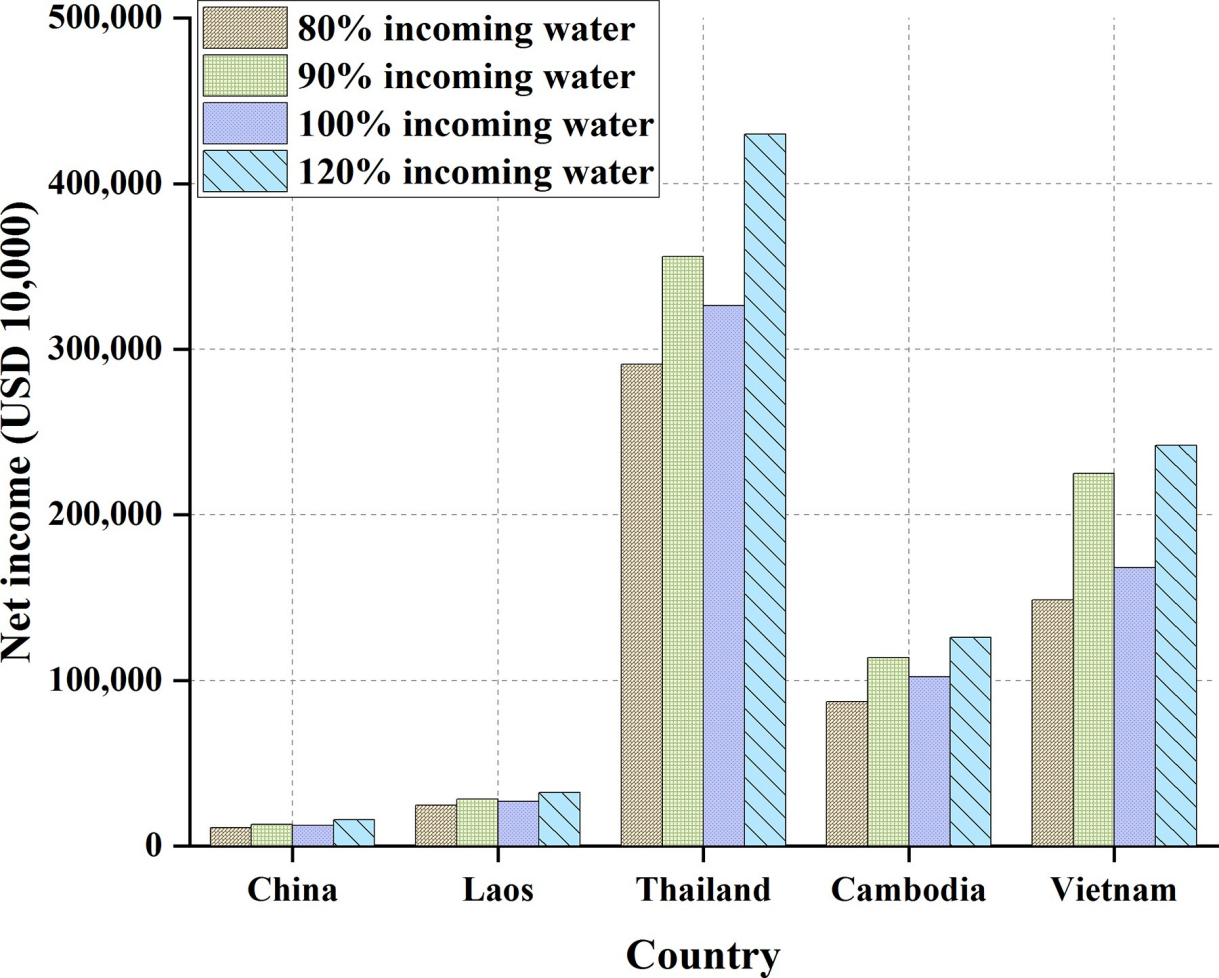
Comparison of five countries’ benefits of independent water resource development.

The overall net benefits of the five countries are summarized and counted. The results of each state’s benefits from the independent water resource development are shown in Table 12 and Fig 18.

**Table 12:** Benefits of independent development of water resources in the five countries.

Countries	80% Inflow/(USD 10,000)	90% Inflow/(USD 10,000)	100% Inflow/(USD 10000)	120% Inflow/(USD 10,000)
China	11,133	13,174	12,525	15,930
Laos	24,737	28,285	26,938	32,436
Thailand	291,044	355,938	326,252	429,994
Cambodia	87,329	113,963	102,146	125,994
Vietnam	148,595	225,073	168,292	242,063

**Fig 19:**
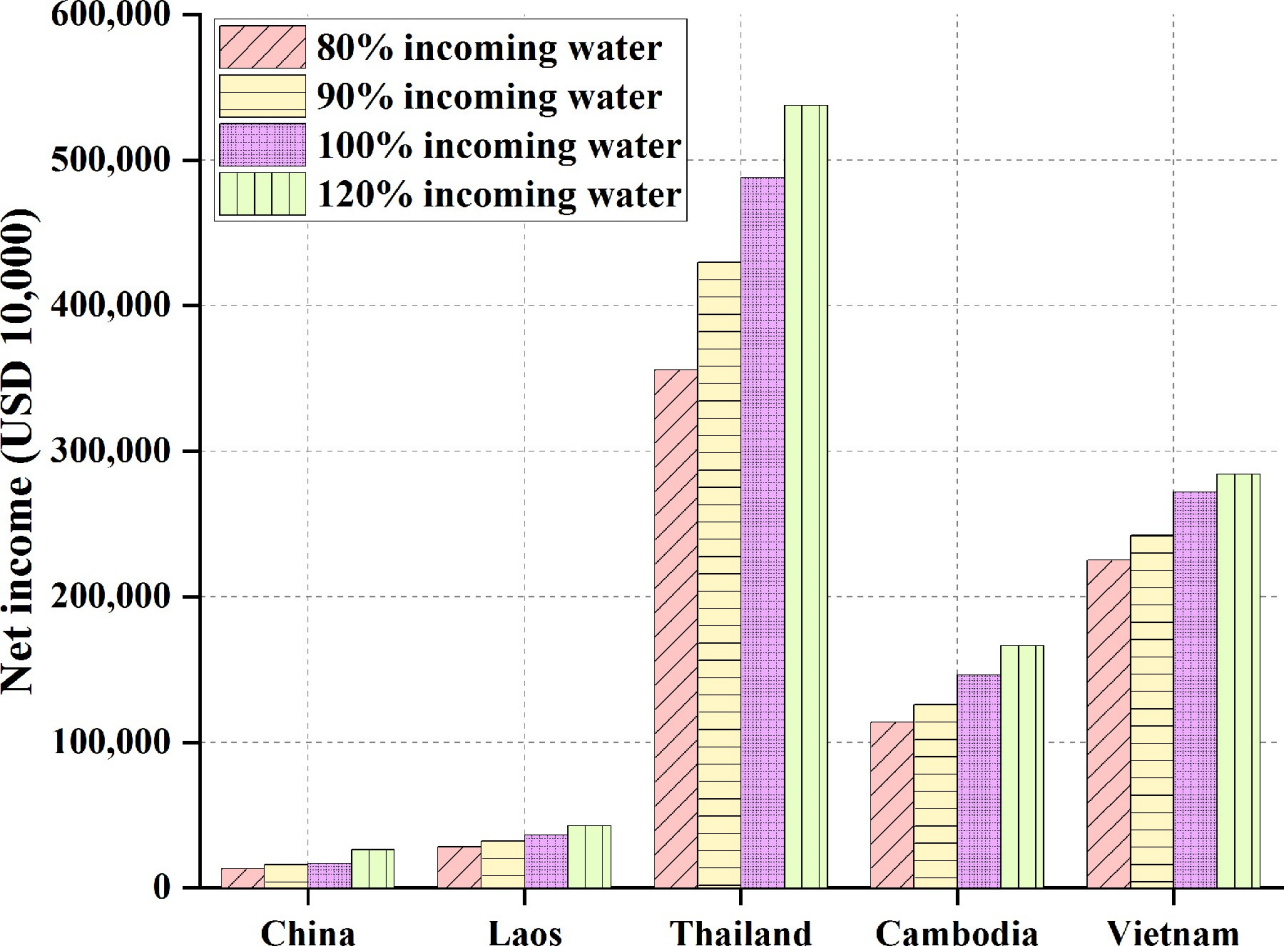
Benefits of each member state under the cooperation of the five countries.

The results of membership benefits under the cooperation of the five countries are shown in Table 13 and Fig 19.

**Table 13:** Each state’s benefits under the cooperation of the five countries.

Countries	80% Inflow/(USD 10,000)	90% Inflow/(USD 10,000)	100% Inflow/(USD 10,000)	120% Inflow/(USD 10,000)
China	13,174	15,930	16,866	26,107
Laos	28,285	32,436	36,353	42,918
Thailand	355,938	429,994	488,120	537,564
Cambodia	113,963	125,994	146,355	166,395
Vietnam	225,073	242,063	271,982	284,389

Figs 18 and 19 demonstrate that the benefit is lower under independent water resources development than that obtained by the five-country cooperation. Meanwhile, China’s irrigation benefit in the Lancang-Mekong River Basin is the lowest among the five countries, suggesting that China’s irrigation demand for the Lancang-Mekong River Basin is relatively small. By comparison, Thailand’s irrigation benefit in the Lancang-Mekong River Basin is the largest, exceeding a substantial level over other countries.

### Result analysis

Here, the PSO algorithm is used to analyze the example and obtain the reference value of the planning results, showing that it is feasible to use the PSO algorithm to obtain the Nash equilibrium of a single scheme game problem. Compared with GA, this algorithm can be directly optimized. The average server occupancy time and average computing waiting time under different tasks are 78.46% and 79.24% lower than the GA algorithm, respectively, showing more efficient computing performance and more suitable for optimizing practical projects. Meanwhile, it can be used for the performance evaluation of emergency water replenishment in cross-border waters. The proposed PSO game model can quickly find the optimal combination among 16 cooperation modes and has guiding value for maximizing the utilization benefits of cross-border water resources. Five countries except Myanmar in the Lancang-Meigong River Basin are taken for the experiment. The benefits of various cooperation in the proportional method and the two-part allocation method of agricultural water use in the five countries are comparatively analyzed using the PSO algorithm and cooperative game model. The results show that when the water inflow of the basin is lower than that in normal years, the two-part matching method plays a significant role in improving the overall benefits of various cooperation. In the year of normal water inflow, there is little difference between the two distribution effects; in the wet years of the basin, the two-part matching method has fewer side effects and is not easy to cause floods. Therefore, the two-part allocation method can be sued for water allocation. In the case of the game with the same number of participants, the total benefits obtained by the game are different. Therefore, each country can choose the trading partner carefully. In the case of cooperation, the benefits of the five countries are significantly higher than those of their separate utilization of water resources. The original Lancang-Mekong cross-border water resources cooperation mechanism is only suitable for the initial development of cooperation. It has improved the economic benefits of countries along the basin during that time. However, as time goes by and under external influence, such as the intervention of foreign governments or the El Nino phenomenon, the traditional cooperation mechanism reveals its disadvantages. Countries in the basin begin to take the water resources cooperation mechanism to seek self-benefits, and the essence of mutually beneficial cooperation has almost disappeared. In this case, there is increasingly tricky cooperation between China and other countries in the Basin. Mutual distrust will also evolve into increasingly acute contradictions and conflicts. Determinedly, China should attach importance to international cooperation, strengthen cooperation and contact with neighboring countries, use the new Lancang-Mekong River cooperation mechanism to assist the rapid advancement of the "Belt and Road Initiative" strategy. In recent years, China has adjusted the original cooperation mode in the Lancang-Mekong River Basin and actively cooperated in hydropower projects with other countries along the basin. In this way, all countries in the Basin have achieved good economic benefits, which has laid a foundation for in-depth cooperation between Lancang-Mekong River Basin countries.

## Conclusion and future works

This paper aims to study the problem of emergency water replenishment in cross-border basins, solve the possible contradictions and conflicts among sharing countries in cross-border basins, and build harmonious international relations based on the basic theoretical knowledge of the PSO algorithm and cooperative game model. Specifically, it analyzes the water consumption and distribution for countries in Lancang-Mekong River Basin. The water consumption and benefits of countries are calculated under 16 cooperation modes. The results show that the PSO algorithm proposed in this paper is more computationally efficient and convenient to operate than other popular algorithms and is more suitable for practical engineering optimization. The algorithm in this paper can quickly obtain the optimal combination of cooperation modes of each country, and the performance is excellent. The total benefits received by the game are different in the case of the same number of participants. Therefore, each country can choose the trading partner carefully. The premise of more incredible cooperation benefits of water rights trading game is that the growth performance of players has excellent heterogeneity. It is suggested that all countries in the basin negotiate to establish a water rights trading mechanism to improve the water use efficiency. Additionally, under the cooperation mode, the benefits of the five countries are significantly higher than those of their respective independent utilization of water resources.

China’s "Belt and Road Initiative" strategy should apply to build a new water resources cooperation model for China’s participation. There is a need for China to expand the resource cooperation of Lancang-Mekong River Basin countries and negotiate to establish a perfect cooperation system based on mutual trust, thereby making up for the defect of simplification of original resource cooperation. Meanwhile, it is necessary to develop corresponding think tanks to ensure the normal development of regional water resources. It can promote water resources cooperation in the Lancang-Mekong River Basin, provide corresponding suggestions for watershed Water resources allocation when necessary, and enhance the overall profits of basin countries’ water resources cooperation. Furthermore, the Lancang-Mekong cooperation framework should be enhanced, and internationally recognized data should be obtained on water resources in the Lancang-Mekong River Basin. Still, there are some shortcomings. The whole experimental process is realized by software simulation, which is different from the actual situation. Moreover, the value of algorithm parameters has not been deeply studied. In addition, this paper only studies the benefits of water utilization and irrigation water in the Lancang-Mekong River Basin and does not explore the benefits of hydropower. The follow-up work will improve the model according to the actual situation and comprehensively consider the hydropower benefits to provide a more effective water resources allocation scheme. The present work aims to provide critical technical support for solving the possible contradictions and conflicts between cross-border basin-sharing countries and building harmonious international relations.

## Supporting information

S1 Data (XLSX)
